# Cross-Linguistic Differences in the Neural Representation of Human Language: Evidence from Users of Signed Languages

**DOI:** 10.3389/fpsyg.2012.00587

**Published:** 2013-01-02

**Authors:** David P. Corina, Laurel A. Lawyer, Deborah Cates

**Affiliations:** ^1^Cognitive Neurolinguistics Laboratory, Center for Mind and Brain, Department of Linguistics, University of California DavisDavis, CA, USA

**Keywords:** aphasia, American sign language, deaf, neurolinguistics

## Abstract

Studies of deaf individuals who are users of signed languages have provided profound insight into the neural representation of human language. Case studies of deaf signers who have incurred left- and right-hemisphere damage have shown that left-hemisphere resources are a necessary component of sign language processing. These data suggest that, despite frank differences in the input and output modality of language, core left perisylvian regions universally serve linguistic function. Neuroimaging studies of deaf signers have generally provided support for this claim. However, more fine-tuned studies of linguistic processing in deaf signers are beginning to show evidence of important differences in the representation of signed and spoken languages. In this paper, we provide a critical review of this literature and present compelling evidence for language-specific cortical representations in deaf signers. These data lend support to the claim that the neural representation of language may show substantive cross-linguistic differences. We discuss the theoretical implications of these findings with respect to an emerging understanding of the neurobiology of language.

## Introduction

Case studies of deaf signing individuals with acquired brain damage and neuroimaging studies of healthy deaf subjects provide evidence for the importance of left-hemisphere systems in the mediation of signed languages. Deaf signers, like hearing speakers, exhibit language disturbances when left-hemisphere cortical regions are damaged and appear to be remarkably spared following right-hemisphere lesions that may nonetheless disrupt non-linguistic visual-spatial functions (Poizner et al., 1987). Neuroimaging studies of deaf signers have generally provided support for this claim (for recent reviews see Campbell et al., 2008; Corina and Spotswood, 2012). However, the fact that emergent linguistic forms in sign languages capitalize upon the affordances and constraints of the visual system rather than the auditory system, and, further, that the production of signs requires a qualitatively different system of articulatory control, leaves open the possibility that there may be divergences and subsequent specializations of the neural systems that underlie the mediation of signed languages. To date, the great majority of articles discussing the neural representation for signed languages have been strongly biased toward reporting the commonalities between the neural representation of speech and sign, and it is only recently that some researchers have begun to call into question the purported close homology between the neural representations of speech and sign (see for example MacSweeney et al., 2008). To understand this state of affairs, it is important to provide a bit of historical background.

It was not until the publication of Sign Language Structure in the 1960’s when William Stokoe and colleagues presented the first formal linguistic analysis of American Sign Language (Stokoe et al., 1965). Prior to this time, the understanding of signed languages within the scientific community was quite misinformed. While William Stokoe’s impact on Linguistics and Education was quite minimal (McBurney, 2001), this work formed a solid base for what was to become a new field of research: Sign Language Linguistics (for a recent exposition, see Sandler and Lillo-Martin, 2006). When the initial psycholinguistic studies of signed languages such as ASL were published (see for example Klima and Bellugi, 1979), they came at a time when there remained numerous misconceptions about the status of signed languages (many of which unfortunately persist today). These early studies helped educate scientists and lay persons alike that signed languages like ASL are not “universal languages,” nor are they primitive forms of communication with limited expressivity, or manual codes made up by educators to represent the words of a spoken language. Few people understood that signed languages are true human languages, albeit expressed in a different signaling modality.

Thus, the pioneering findings by Bellugi and colleagues were indeed news. They discovered, for example, that language acquisition milestones for infants learning a signed language from deaf parents as a native language paralleled native spoken language milestones, that signs are used as a basis for memory codes used by deaf signers, and that signed languages, like spoken languages, can be analyzed as compositional forms that exhibit linguistic complexity and have systematic means for marking morphological and syntactic properties (see Klima and Bellugi, 1979). As neuropsychological studies of deaf signers began to appear, once again there was a perceived need to convincingly demonstrate that sign languages were true human languages. Emerging data from studies of sign language aphasia and neuroimaging further showed that demonstrably similar brain areas are used during sign and spoken language processing. The reporting and initial interpretations of this exciting behavioral and neurological data from sign languages used by deaf persons led many to believe that language modality had little influence on the core properties of linguistic function; the processes and neural regions underlying sign and speech functions were considered largely isomorphic, a testament to the integrity of the neurobiology of the human capacity for language. Indeed, any research that suggested otherwise was openly challenged as being a methodological anomaly (cf. Hickok et al., 1998). Fortunately, as the message about the status of signed languages as legitimate human languages became more widespread, researchers began to question the lore of the otherwise neat and seemingly inseparable homologies that had been constructed to describe the convergence of neural systems mediating signed and spoken languages. In addition, the growing awareness that spoken language may call upon both left- and right-hemisphere resources made the idea that human languages may have bi-hemispheric representation seem less challenging. Given the advances in the scientific discourse of the language sciences, it is worth considering the possibility that the diversity of linguistic forms exhibited by the world’s language in fact have real processing consequences, and these processing differences have identifiable neural consequences. The stark contrast between sign and speech language modalities provides a privileged vantage point to begin such explorations.

In this paper, we explore three specific examples that suggest qualitative differences in neurofunctional instantiation of signed languages. We first consider the possibility that hemispheric specialization for signed languages and spoken languages may differ; in particular, we review mounting evidence that unique right-hemisphere resources may be required for competency in ASL in a manner that is not observed for spoken languages. Second, we consider the evidence that, within the left-hemisphere, the cortical organization for signed languages may differ in substantive ways from what is commonly observed in spoken languages. Finally we consider evidence that suggests that the requirements for the fluent expression of a signed language entail unique motoric constraints, and present evidence for sign-language-specific linguistic disorders. We then consider the implications of these data for our understanding of the neurobiology of human language.

## Hemispheric Specialization for Language

Two of the most widely established facts of human neuroscience are that the two cerebral hemispheres exhibit differential specialization for cognitive functions and that the left-hemisphere perisylvian regions play a predominant role in processing of spoken language. In expressing these differences, it was common to claim that some predefined components of language processing (for example: phonology, semantics, and syntax) represent “core-linguistic functions,” which are somehow more elemental in status, while everything else (for example, properties of discourse and pragmatics), are simply “extra-linguistic functions.” More recently we can observe a re-characterization of the neural representation of speech which claims the left temporal-lobe exhibits more domain-specific mechanisms, while the right-hemisphere exhibits domain-general mechanisms (McGettigan and Scott, 2012). Fortunately, concurrent with this new characterization has been the development of sophisticated neuroimaging techniques and signal manipulations which can help elucidate the essence of linguistic specificity, and the determination of hemispheric specialization remains an active area of research (see McGettigan and Scott, 2012 for a recent review). In addition, there are refreshing developments in the efforts to understand the contributions of both hemispheres in speech perception (see for example Scott et al., 2000; Zatorre and Belin, 2001; Belin and Zatorre, 2003; Poeppel, 2003; Ben Shalom and Poeppel, 2008). One of the most important developments and particularly germane to the present paper is the acknowledgment that understanding the neural mechanisms of speech processing requires an appreciation of the intimate coupling between acoustic perception and the articulatory system that gives rise to speech sounds (McGettigan and Scott, 2012). Given this state of affairs it is certainly worth considering whether the frank modality differences between speech and signed language lead to qualitative differences in neural systems mediating the alternative forms of human communication.

The fact that *left-hemisphere* perisylvian regions appear critical for the fidelity of sign language expression has been well-established and is uncontroversial (Poizner et al., 1987; Corina, 1998a; Hickok et al., 2002; MacSweeney et al., 2008). However, an unresolved issue concerns the role of the *right-hemisphere* in the mediation of ASL and indeed other naturally occurring signed languages such as British Signed Language (MacSweeney et al., 2002b). Data from both lesion studies and neuroimaging indicate that right-hemisphere parietal regions may play a prominent role in the processing of signed languages. A critical question is whether this reliance on the right-hemisphere represents domain-specific linguistic mechanisms or domain-general visual-spatial processing, including processes related to human action understanding.

Initial studies reported that signers with damage to the right-hemisphere had impaired visual-spatial deficits but well-preserved language skills (Poizner et al., 1987). However, as further studies appeared, it became clear that right-hemisphere damage in users of signed languages also disrupted the meta-control of language use, which resulted in disruptions of discourse abilities (Hickok et al., 1999). This finding is similar to those suggesting right-hemisphere damage in users of spoken language impacts so-called extra-linguistic functions, such as the interpretation of metaphors and humor (Brownell et al., 1990; Kaplan et al., 1990; Rehak et al., 1992). Yet more interesting are the indications that right-hemisphere lesions may fundamentally disrupt aspects of syntactic processing in signed languages. The initial reports of this possibility are in fact stated in Poizner et al. (1987), who note that while left-hemisphere damage commonly results in disturbances of syntactic processing of ASL, signers with right-hemisphere damage also exhibited syntactic comprehension problems. For example, subjects S. M. and G. G. [right-hemisphere damaged (RHD) subjects tested by Poizner et al., 1987] performed well below controls on two tests of spatial syntax. Indeed, as the authors point out, “right lesioned signers do not show comprehension deficits in any linguistic test, other than that of spatialized syntax.” Poizner et al. (1987) speculated that the perceptual processing involved in the comprehension of spatialized syntax involves both left- and right-hemispheres, and that certain critical areas of both hemispheres must be relatively intact for accurate performance.

Additional evidence for right-hemisphere contributions for aspects of sign comprehension come from the consideration of the way in which signed languages routinely capitalize upon the postural properties of the body, the manual articulators, and the spatial affordances of the visual system to convey complex meanings including grammatical roles (such as subject/object), prepositional meaning, locative relations, and speaker viewpoint in ways that may not have direct parallels in spoken languages. For example, certain classes of sign forms have been described as depictive. That is, some verbs have, in addition to their usual function as verbs, the ability to depict the event they encode (Liddell, 2003). As described in Dudis (2004, 2007) the contrast between a non-depicting agreement verb, such as GIVE, versus a depicting verb, such as HAND-TO, illustrates some of these property differences. The handshape and the movement in the verb GIVE is not conditioned by the object being given (though the direction of the movement may be conditioned by grammatical structure); in contrast, the verb HAND-TO can be used to describe only the transfer of objects that can be held between the thumb and the four fingers of the handshape – a paper document or credit card, but certainly not a kitchen blender. This is one way that the verb’s iconicity constrains its usage. Additionally, the palm’s continuously upward orientation and the path of the hand created via the elbow emulate the physical motion of the transfer event. Dudis further suggests that it is not solely the morphophonological differences in the handshape that differentiates these forms, but rather the verb’s ability to portray a dynamic and visual representation of transfer, which is a *demonstration* rather than plain *description*.

In a similar fashion, spatial prepositional relations between objects such as “on, above, under,” etc., can be conveyed via the depiction of the physical relation itself rather than encoded by a discrete lexical item. For example, an ASL translation of the English sentence “The pen rests on a book” may, in part, involve the use of the two hands whereby one hand configuration with an outstretched finger (representing the pen) is placed on the back of a flat open hand (representing the book). This configuration encodes the spatial meaning “on” but without the need for a conventional lexical preposition corresponding to “on.”

Many signed languages express locative relationships and events in this manner and have discrete inventories of highly productive grammatical forms, typically referred to as classifiers or classifier predicates, which participate in these depictive constructions. While the use of these forms constitutes a major contrastive linguistic function in signed languages (see Emmorey, 2003 and papers therein) their theoretical status remains a point of vibrant discussion and debate (for contrastive views see Liddell, 2003; Sandler and Lillo-Martin, 2006).

This controversy, however, has important implications for our understanding of the neurolinguistics of signed languages, as several studies have found differential disruptions in the use and comprehension of sentences that involve “depictive” forms. For example, Atkinson et al. (2005) conducted a group study of LHD and RHD signers of British Sign Language (BSL). Their tests included single-sign and single-predicate verb constructions (e.g., THROW-DART), simple, and complex sentences that ranged in argument structure and semantic reversibility, locative constructions encoding spatial relationships and lexical preposition constructions, and a final test of classifier placement orientation and rotation. Their findings indicated that left-hemisphere damaged (LHD) BSL signers relative to elderly control subjects exhibited deficits on all comprehension tests. RHD signers did not differ from controls on single-sign and single-predicate verb constructions or sentences that ranged in argument structure and semantic reversibility. RHD signers (like LHD signers), however, were impaired on tests of locative relationships expressed via classifier constructions and on tests of classifier placement orientation and rotation.

One interpretation offered for this pattern of responses is that the comprehension of these classifier constructions requires not only intact left-hemisphere resources but intact right-hemisphere visual-spatial processing mechanisms. That is, while both LHD and RHD signers show comprehension deficits, the RHD signers’ difficulties stem from more general visual-spatial deficits than linguistic malfunctions *per se*. Yet there has been little attempt to specify the exact nature of the visual deficits that give rise to these language comprehension problems. An important question is whether these general visual-spatial deficits should be deemed “extra-linguistic” or, rather, be considered a linguistic property of signed languages. For example, Atkinson et al. (2005) suggest “the deficits stem from the disruption of processes which map non-arbitrary sign location on the real-world’s spatial position.” However, depictive forms are not only used to refer to real-world events, but also to imaginary and non-present events as well. In ASL for example, one may use distinct spatial locations to establish and contrast two theoretical ideas, using separate signing space and references to these spatial locations to convey semantic and causal relationships between these theoretical constructs. The broader point is whether *aphasic* deficits should be solely defined as those that have clear homologies to the left-hemisphere maladies evidenced in spoken languages, or whether the patterns observed in the disruption of signed languages will force us to reconsider the conception of linguistic deficits (see also MacSweeney et al., 2008 for discussion). An additional avenue of inquiry to further illuminate the processing requirements of spatial forms in signed language will come from a more systematic comparison between the role of comprehension of these spatial forms compared with their production (Emmorey et al., 2004).

Neuroimaging studies of deaf signers raise additional questions concerning the role of the right-hemisphere in sign language processing. Studies of sentence processing in signed languages have repeatedly reported left-hemisphere activations that parallel those found for spoken languages. These activation patterns include inferior frontal gyrus (including Broca’s area and insula), precentral sulcus, superior, and middle temporal cortical regions, posterior superior temporal sulcus, angular gyrus, and supramarginal gyrus (e.g., Neville et al., 1998; Petitto et al., 2000; MacSweeney et al., 2002a, 2006; Newman et al., 2002; Lambertz et al., 2005; Sakai et al., 2005). Activations in these regions are often found in studies of auditory language processing (Blumstein, 1994; Schlosser et al., 1998; Davis and Johnsrude, 2003).

However, in addition to the more familiar left-hemisphere activations, studies of sentence processing in signed languages have also noted significant right-hemisphere activation. For example, activations in right-hemisphere superior temporal, inferior frontal, and posterior-parietal regions have been reported (e.g., Neville et al., 1998; MacSweeney et al., 2002a, 2006; Newman et al., 2002). The question of whether these patterns of activation are unique to sign has been a topic of debate (see for example: Corina et al., 1998; Hickok et al., 1998), as studies of auditory and audio-visual speech have observed right-hemisphere activations that appear similar to those reported in signing (e.g., Schlosser et al., 1998; Davis and Johnsrude, 2003). More recent evidence suggests that the right-hemisphere lateral superior temporal activations may not be sign-specific. Capek et al. (2004), for example, showed that for monolingual speakers of English, audio-visual English sentence processing elicited not only left-dominant activation in language regions, but also bilateral activation in the anterior and middle lateral sulcus. A direct comparison of audio-visual speech processing in hearing non-signers and BSL processing in deaf signers revealed highly overlapping regions of activation including bilateral activation of the temporal-lobes under each linguistic condition (MacSweeney et al., 2002a). Previous studies have shown that right-hemisphere superior temporal regions are sensitive to facial, and especially mouth, information (Puce et al., 1998; Pelphrey et al., 2005). It is well known that deaf signers focus attention on mouth regions while attending to signs (Muir and Richardson, 2005). Thus, these studies suggest that lateral temporal activation patterns are not sign-specific but are likely indexing aspects of the recognition of physical human forms.

In contrast to the common temporal-lobe physical-form regions, neuroimaging data further suggests that right posterior-parietal and posterior-temporal regions may play a special role in the mediation of signed languages (Bavelier et al., 1998; Newman et al., 2002). In a series of studies, deaf and hearing native signers, hearing non-signers, and hearing late learners of ASL viewed sign language sentences contrasted with sign gibberish. Deaf and hearing native signers showed significant activation in right-hemisphere posterior-parietal and posterior-temporal regions, including a homolog of the posterior-temporal Wernicke’s area. These activation patterns were not seen in non-signers, nor were they observed in hearing late learners of ASL. A group analysis of native and late-learning hearing signers confirmed that the right angular gyrus was found to be active only when hearing native users of ASL performed the task. When hearing signers who learned to sign after puberty performed the same task, the right angular gyrus activation was not observed. Newman et al. (2002) argued that the activation of this neural region during sign language perception might be a neural signature of sign language acquisition during the critical period for language. Many researchers have speculated that right-hemisphere parietal activation in signers is associated with the linguistic use of space in sign language (Bavelier et al., 1998; Newman et al., 2002), and recent studies have sought to clarify the contributions of spatial processing in ASL and BSL to observed right-hemisphere activations.

MacSweeney et al. (2002b), for example, compared the role of parietal cortices in an anomaly detection task in BSL. They tested deaf and hearing native signers in a paradigm that utilized BSL sentence contexts that either made use of topographic signing space or did not require topographic mapping. Across both groups, comprehension of topographic BSL sentences recruited the left parietal [BA 40 and superior parietal lobule (SPL)-BA 7] and bilateral posterior middle temporal cortices to a greater extent than did non-topographic sentences. Activation during anomaly detection in the context of topographic sentences was maximal for left-hemisphere inferior parietal lobule (IPL) in skilled signers. MacSweeney et al. (2002b) suggest these activation patterns may be related to requirements for spatial processing of hands, as studies of non-signers have observed left IPL activation in response to imagery of hand movements and hand position (Kosslyn et al., 1998; Gerardin et al., 2000; Hermsdorfer et al., 2001). A second left parietal region in these studies, BA 7, is suggested to reflect similar mechanisms of hand or finger movement (e.g., Weeks et al., 2001). An alternative explanation suggests that neural regions for action processing may have been more prevalent during the topographic sentences. Several researchers have observed SPL activation in response to human action and action vocabulary more generally (Damasio et al., 2001; Grezes and Decety, 2001; Hauk et al., 2004; Pulvermüller et al., 2005).

Importantly, and similar to the findings reported by Newman et al. (2002), native deaf signers in the MacSweeney et al. (2002b) study did show activation in the right angular gyrus (BA 39). Parietal activation in the hearing native signers, however, was modulated by accuracy on the task, with more accurate subjects showing greater activation. This finding suggests that proficiency, rather than age of acquisition, may be a critical determinant of right-hemisphere engagement. Importantly, activation in right-hemisphere temporal-parietal regions was specific to BSL and was not observed in hearing non-signers watching audio-visual English translations of the same sentences.

Further evidence of the differential contributions of cerebral in sign comprehension is found in the study reported by Capek et al. (2009). These researchers used electrophysiological recording to assess the brain systems mediating semantic and syntactic aspects of ASL processing. In this study deaf native signers watched ASL sentences that were either grammatically well-formed or grammatically anomalous. The grammatical anomalies included violations of semantic expectancies and two different syntactic violations. In both cases the syntactic violations entailed the misuse of accepted conventions of spatial syntax. In one case, “reversed” verb-agreement violations were formed by reversing the direction of the verb such that the verb moved toward the subject instead of the object. The second grammatical violation made use of “unspecified” verb-agreement violations, which were created by directing a spatial-agreement verb toward a location in space that had not been defined previously as the subject or object.

As expected, semantically anomalous ASL sentences elicited an N400 (300–875 ms) relative to control sentences. This N400 response was broadly distributed over posterior regions in a pattern widely consistent with studies of semantic violations in aural-oral languages. Syntactic violations elicited an anterior negativity followed by a widely distributed P600; however, compelling differences in the distribution of this early negativity were noted. Reversed verb-agreement violations elicited an early anterior negativity (140–200 ms) that was largest over the lateral sites of the left-hemisphere, i.e., a Left Anterior Negativity similar to what has been found in studies of syntactic violations in spoken languages (Friederici, 2002). In contrast, unspecified verb-agreement violations, relative to canonical sentences, elicited an anterior negativity (200–360 ms) that was largest over the *right* lateral frontal sites. As with the reversed verb-agreement violations, sentences containing unspecified verb-agreement anomalies also elicited a broadly distributed P600 that was larger over the left-hemisphere.

Taken together, these ERP data suggest that there are distinct brain systems mediating semantic and syntactic processing in ASL, as has been observed for spoken languages (Kutas and Hillyard, 1980, 1984; Neville et al., 1991). Importantly, the data suggests great similarities in systems mediating semantic processing in spoken and signed language (see also Neville et al., 1997). In contrast, the syntactic violations evoked left anterior negativity in the case of the reversed verb-agreement sentences, and unexpectedly a *right* anterior negativity for unspecified verb-agreement violations. Capek et al. (2009) suggest that the unspecified verb-agreement violations likely place different demands on the system involved in processing spatial syntax than the reversed verb-agreement violations. The unspecified verb-agreement violations refer to a spatial location at which no referent had previously been located. In these cases, the viewer is forced to either posit a new referent whose identity is unknown (and will perhaps be introduced at a later time in the discourse) or infer that the intended referent is one that was previously placed at a different spatial location. Either way, different processing is required compared with the reversed violations. Capek et al. (2009, p. 8787) state “The results clearly implicate distinguishable neural subsystems involved in the processing of ‘spatial syntax’ in ASL depending on the processing demands, and suggest a more complex organization for the neural basis of syntax than a unitary ‘grammatical processing’ system.”

Thus, when one closely examines the data from neuropsychological case studies of deaf signers with left- and right-hemisphere damage, neuroimaging data of ASL and BSL comprehension, and electrophysiological studies of syntactic processing in ASL, a consistent story begins to emerge. Specifically, it appears that the comprehension of particular grammatical constructions, including aspects of syntax and classifier-predicate constructions in signed languages, requires right-hemisphere resources and, as suggested by the neuroimaging data, right posterior-parietal resources in particular, in a manner that is not generally reported for the comprehension of spoken languages (see for example Emmorey et al., 2002).

If we accept this claim, then these data raise important theoretical questions for our understanding of the biological determination of neural regions required for human language processing. However, before we accept this claim, we need to be careful in our assessment. There are at least three logical alternative possibilities that may account for appearance of right-hemisphere resources during aspects of grammatical and classifier processing in naturally occurring signed languages. First, one may assume that the right-hemisphere involvement is not specifically “linguistic” in nature, but rather reflects general visual-spatial processing. That is to say, the observed activations (and impairments) simply reflect general visual-spatial resources that are required for processing of perceived human movements. Because sign languages require the registration of such visual-spatial information, and right-hemisphere parietal regions normally participate in these functions, it stands to reason that we may observe activation of these regions during sign processing and deficits in processing when these general-purpose regions are damaged. Under this view, we don’t observe activations for spoken languages as such requirements for the registration of human movements are not required.

A second logical possibility is that the right-hemisphere involvement does reflect linguistic processing; however, this processing is not specific to signed languages. It is simply the case that we have yet to systematically identify the proper grammatical constructions in spoken languages that trigger the involvement of this region. For example, perhaps there are languages with complex case-marking systems which require registration of the spatial-temporal ordering of morphemes for determining subject and object relationships; or, perhaps, appreciation of specific spatial-preposition constructions whose processing dynamics have yet to be fully explored.

A third possibility is that the right-hemisphere activation reflects general visual-spatial processes and is used in the perception of human movements that underlie both sign and speech. As discussed above, this account has been evoked to explain the lateral temporal activation seen during the processing of audio-visual speech and signed language, but fails to account for the right posterior-parietal activations reported in Newman et al. (2002). We consider further implications of these findings below, but first we examine the possibility that even within the left-hemisphere there may be observable language modality conditioned effects that differentiate spoken and signed languages.

## Left-Hemisphere Specialization

Signers with left-hemisphere posterior lesions evidence fluent sign aphasia with associated comprehension deficits. There is, however, controversy in regards to anatomical regions that may give rise to comprehension problems in spoken and signed languages. In an effort to examine the role of the temporal lobe in ASL comprehension deficits, Hickok et al. (2002) compared the sign language comprehension abilities of LHD and RHD signers. Signers with left-hemisphere posterior temporal lobe damage were found to perform worse than any other group, exhibiting significant impairments on single-sign and sentence performance as accessed by an ASL translation of the token test (De Renzi and Vignolo, 1962). While the authors emphasize the involvement of the damaged temporal lobe in these comprehension deficits, though not discussed, in all cases the lesions additionally extended into the parietal lobe. Thus is it unclear whether sign language comprehension deficits require impairment to the temporal lobe only, the parietal lobe only, or some combination thereof.

It is noteworthy that the cases described by Chiarello et al. (1982) and Poizner et al. (1987) and the case study described by Corina et al. (1992), exhibited fluent aphasia with severe comprehension deficits. Lesions in these case studies did not occur in cortical Wernicke’s area proper, but rather involved more frontal and inferior parietal areas. In these cases, lesions extended posteriorly to the supramarginal gyrus. This is interesting, as lesions associated with the supramarginal gyrus alone in users of spoken language do not typically result in severe speech comprehension deficits. These observations have led some to suggest that sign language comprehension may be more dependent than speech on left-hemisphere inferior parietal areas, that is, regions associated with somatosensory and visual motor integration (Leischner, 1943; Chiarello et al., 1982; Poizner et al., 1987; Corina, 1998a,b), while spoken language comprehension might depend more heavily on posterior temporal lobe association regions whose input includes networks intimately involved with auditory speech processing. Currently the lack of an adequate number of well-described case studies limits our ability to revolve this controversy. However, as researchers begin to provide more rigorous behavioral assessments of sign language comprehension defects following right and left-hemisphere damage (see for example, Atkinson et al., 2005), coupled with the ability to attain high-resolution structural images of these subjects, for instance using voxel-based lesion symptom mapping techniques (Bates et al., 2003), more objective assessments of functional-anatomical relationships of the posterior temporal and inferior parietal lobes will be possible.

## ASL Paraphasia

Sign language breakdown following left-hemisphere damage is not haphazard, but affects independently motivated linguistic categories (e.g., semantics, phonology, morphosyntax). This observation provides support for viewing aphasia as a unique and specific cognitive deficit rather than as a subtype of a more general motor or symbolic deficit. An example of the systematicity of paraphasic errors across signed and spoken languages can be found in Corina (2000). These include examples of both semantic paraphasia and formational (phonemic) paraphasias. The formational paraphasias elucidate the differential vulnerability of the sublexical structures (i.e., handshape, movement, articulatory location) of signed languages. These errors demonstrate how functionally similar language categories (e.g., “major class segments”) may be selectively vulnerable to impairment. While the surface manifestations differ, the underlying disruption may be related to a common abstract level of representation.

An unusual case of sign paraphasia that does not have a clear mapping to patterns observed in spoken language is reported by Hickok et al. (1996). A life-long signer, R. S., suffered an infarct to the left frontal operculum. Neurological examination revealed an initial expressive aphasia that largely resolved with lingering problems of word-finding and frequent phonemic paraphasia. Particularly noteworthy is the nature of these errors which, in contrast to the handshape errors previously described, demonstrate the way in which a language’s modality may uniquely influence the form of the linguistic deficit – in this case, an articulatory impairment with no clear parallels to spoken language disruption. Signed languages, unlike spoken languages, require coordinated control of both hands. The possibility of differential programming of two potentially independent articulators may place qualitatively different demands on the on the linguistic system. In the absence of limb apraxia, R. S. exhibited paraphasia restricted to two-handed signs. For example, on signs that require two hands to assume different handshapes and/or to move independently, R. S. would incorrectly fail to move one of her hands. In other cases, on signs where the appropriate movement of one hand was relative to the position of the other, R. S. might produce an incorrect relation movement in the sense that the movement itself was correct, but it was not carried out correctly with respect to the spatial relationship to the other hand. In addition, during one-handed signing, she exhibited mirroring movement and handshape of the dominant hand on the non-dominant hand that was somewhat reduced in degree of movement. Such mirroring was not seen during non-linguistic movement and was qualitatively different from mirror movements effecting distal movement sometimes seen in cases of hemiparesis (Hickok et al., 1996). While acknowledging the limitations of rare single case studies, we hold that this case is important for our understanding of the neurobiology of language as it indicates that the modality and/or form of a human linguistic system may place unique demands on the neural mediation and implementation of language. These errors can be taken as evidence for selective language-form-specific linguistic impairment.

## Concluding Remarks

In summary, we have considered three specific instances where data from users of signed languages suggests that competencies in sign language use may require participation of neural resources not typically encountered for the processing of spoken language. First, we examined the mounting evidence that the right-hemisphere parietal regions may be required for some aspects of sign comprehension, especially in the context of specialized syntax, as well as in the appreciation of classifier forms. We then considered evidence for the claim that left posterior temporal regions are necessary for sign comprehension. While research shows that left temporal regions do impact sign language comprehension, we noted that research to date has not properly excluded the contributions of left parietal regions in sign language comprehension impairment, thus undermining the claims for isomorphic functions of left temporal lobes in the mediation of all human languages, spoken or signed. Finally, we briefly recounted the unusual presentation of linguistic impairment in case of signer R. S., whose language errors are limited to linguistic productions of two-handed ASL signs, here again providing evidence that specialized neural resources are required to implement articulatory demands of a signed language such as ASL.

There are important theoretical issues raised by these data. Principally, the data suggests a conflict between an amodal account of language processing, in which one considers brain areas responsible for language to be independent of language modality, versus a modality-influenced view of language, whereby one acknowledges that there may be properties required for the execution and understanding of language forms that vary as a function of linguistic form and structure. As stated previously, language researchers are increasingly cognizant of the fact that the neural organization reflects the intimate coupling between language perception and production. In the present case, we have considered the differences and similarities between the neural processing of spoken and signed languages. Some scientists will likely conclude that this represents an extreme situation with a tendency to outright dismiss the validity of the comparisons. However, if we acknowledge that both signed and spoken languages are true manifestations of the capacity for human language, one must consider the possibility that specialized and language-specific neural mechanisms may be required for competency. Generally, researchers tend to avoid this latter position as it leads toward the proverbially slippery slope. If we acknowledge language-specific neural specialization, might we further expect to see cases of language specificity across the diverse forms of spoken languages? The broader point is whether aphasic deficits should be solely defined as those that have clear homologies to the left-hemisphere maladies that are evidenced in spoken languages, or whether the existence of signed languages will force us to reconsider the conception of linguistic deficits such as aphasia and open the possibility that there may be multiple ways in which the human brain may manifest linguistic abilities.

## Conflict of Interest Statement

The authors declare that the research was conducted in the absence of any commercial or financial relationships that could be construed as a potential conflict of interest.
